# Healthcare costs of patients on different renal replacement modalities – Analysis of Dutch health insurance claims data

**DOI:** 10.1371/journal.pone.0220800

**Published:** 2019-08-15

**Authors:** Sigrid M. Mohnen, Manon J. M. van Oosten, Jeanine Los, Martijn J. H. Leegte, Kitty J. Jager, Marc H. Hemmelder, Susan J. J. Logtenberg, Vianda S. Stel, Leona Hakkaart-van Roijen, G. Ardine de Wit

**Affiliations:** 1 Centre for Nutrition, Prevention and Health Services, National Institute of Public Health and the Environment, Bilthoven, The Netherlands; 2 Department of Medical Informatics, Amsterdam UMC, University of Amsterdam, Amsterdam Public Health Research Institute, Amsterdam, The Netherlands; 3 Erasmus School of Health Policy & Management (ESHPM), Institute for Medical Technology Assessment (iMTA), Erasmus University, Rotterdam, The Netherlands; 4 Dutch Renal Registry Renine, Nefrovisie Foundation, Utrecht, The Netherlands; 5 Department of Internal Medicine, Diakonessenhuis Utrecht, Utrecht, The Netherlands; 6 Juliuscentre for Health Sciences and Primary Care, University Medical Centre Utrecht, Utrecht, The Netherlands; University of Verona, ITALY

## Abstract

**Background:**

The aim of this study is to present average annual healthcare costs for Dutch renal replacement therapy (RRT) patients for 7 treatment modalities.

**Methods:**

Health insurance claims data from 2012–2014 were used. All patients with a 2014 claim for dialysis or kidney transplantation were selected. The RRT related and RRT unrelated average annual healthcare costs were analysed for 5 dialysis modalities (in-centre haemodialysis (CHD), home haemodialysis (HHD), continuous ambulatory peritoneal dialysis (CAPD), automated peritoneal dialysis (APD) and multiple dialysis modalities in a year (Mix group)) and 2 transplant modalities (kidney from living and deceased donor, respectively).

**Results:**

The total average annual healthcare costs in 2014 ranged from €77,566 (SD = €27,237) for CAPD patients to €105,833 (SD = €30,239) for patients in the Mix group. For all dialysis modalities, the vast majority (72–84%) of costs was RRT related. Patients on haemodialysis ≥4x/week had significantly higher average annual costs compared to those dialyzing 3x/week (Δ€19,122). Costs for kidney transplant recipients were €85,127 (SD = €39,679) in the year of transplantation and rapidly declined in the first and second year after successful transplantation (resp. €29,612 (SD = €34,099) and €15,018 (SD = €16,186)). Transplantation with a deceased donor kidney resulted in higher costs (€99,450, SD = €36,036)) in the year of transplantation compared to a living donor kidney transplantation (€73,376, SD = €38,666).

**Conclusions:**

CAPD patients have the lowest costs compared to other dialysis modalities. Costs in the year of transplantation are 25% lower for patients with kidneys from living vs. deceased donor. After successful transplantation, annual costs decline substantially to a level that is approximately 14–19% of annual dialysis costs.

## Introduction

End-stage renal disease (ESRD) is ranked among the top 20 leading causes of decrease in quality of life and loss of life years, and has one of the highest disease burdens worldwide [1, 2]. In the Netherlands on January 1^st^ 2015, 16,277 people were dependent on renal replacement therapy (RRT) with an annual incidence of approximately 2,000 patients [3]. Although in the Netherlands the incidence rate of RRT has stabilized, the number of prevalent patients continues to rise due to a relatively high number of renal transplantations [4]. This implies that the economic burden of RRT treatment increases as well. Healthcare systems face a major challenge as a considerable amount of the often limited healthcare budget is spent on RRT [5]. According to the National Institute of Public Health and the Environment (RIVM), the total healthcare cost for chronic renal failure was 800 million Euros in 2011 [2]. As the vast majority of these costs is related to RRT, this implies that 1% of the national healthcare budget of the Netherlands was spent on 0,1% of the population [6].

RRT has always served as a classical example of lifesaving treatment with very high per person costs and this essentially has not changed over the past decennia. However, comprehensive cost estimates of RRT in the Netherlands are based on a study from the 1990’s[6]. More recent studies only incorporated one or a few RRT modalities [7, 8]. Also, recent developments, such as living-donor related kidney transplantation and high-frequency dialysis, necessitate a comprehensive costing study that includes such new therapeutic possibilities.

Several European studies have recently used health insurance claims to investigate national healthcare expenditures related to RRT [9–12]. Dutch health insurance claims contain details on expenditures and treatment of different RRT modality and enable to perform a comprehensive study on healthcare expenditure of patients on different RRT modalities with a nationwide coverage.

The aim of this study is to provide detailed estimates of the average annual costs per patient for seven RRT modalities. Besides distinguishing between dialysis therapies, we also include transplantation costs by source of kidney donor, living or deceased, and haemodialysis (HD) costs by frequency of dialysis.

## Materials and methods

### Data source

In the Netherlands healthcare insurance is obligatory; almost all citizens have a healthcare insurance [13]. Vektis collects and manages claims data of all Dutch health insurance companies. These claims are related to all healthcare procedures covered by the Health Insurance Act, including the costs of compulsory co-payments [14]. The Vektis database covers 99% of insured people living in the Netherlands and contains demographic information, including sex, year of birth and date of death. To ensure privacy, Vektis pseudonymised the persons’ national identification number and allowed data access only in a secured environment. All authors only had access to de-identified data. For the use of this national data, permission of all contributing insurance companies was provided (S1 File).

### Study population

**Fig 1:**
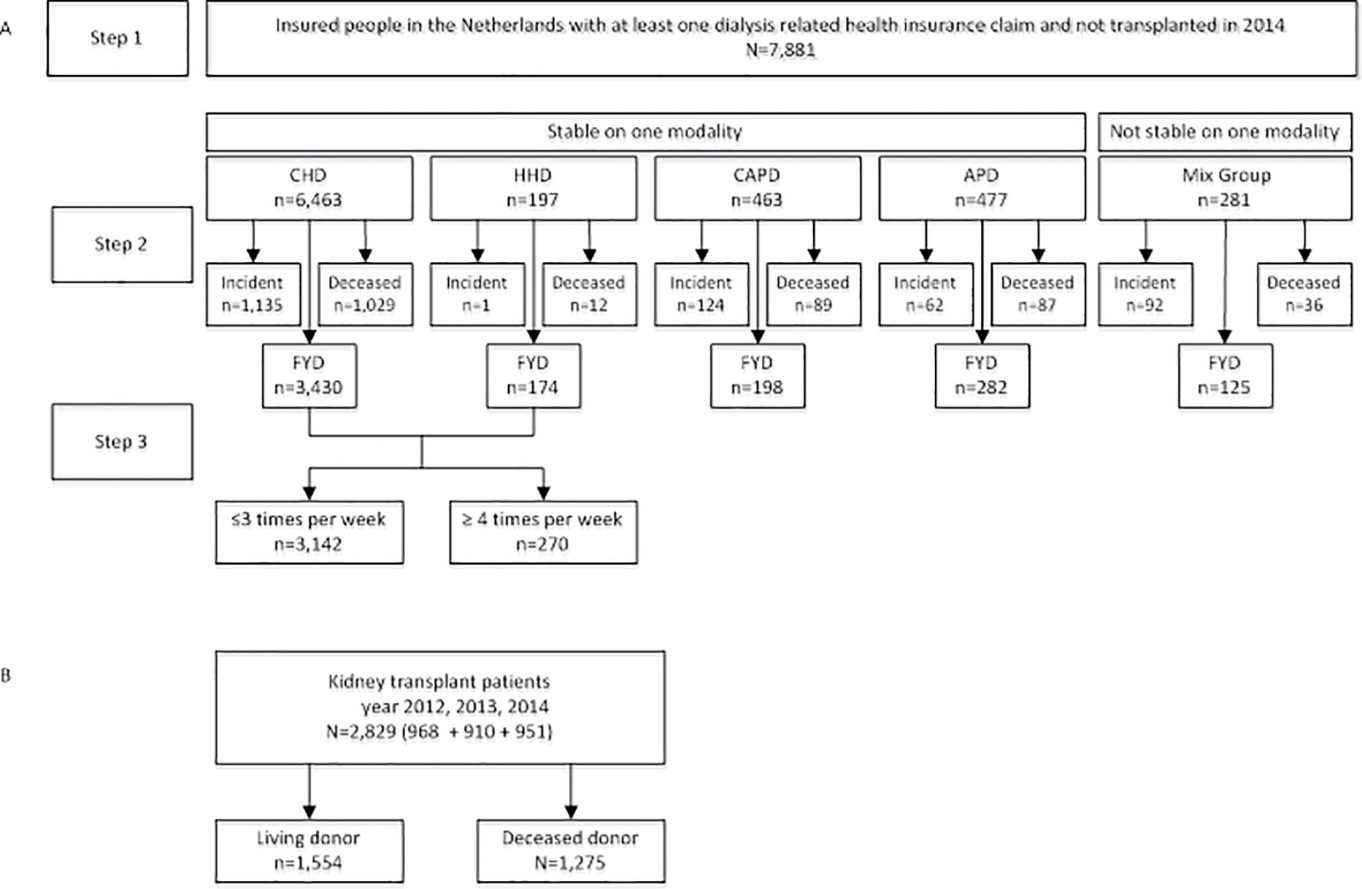
Classification of RRT modalities. Note: Fig 1A: Classification of dialysis patients; CHD = Centre Haemodialysis; HHD = Home Haemodialysis; APD = Automated Peritoneal Dialysis; CAPD = Continuous Ambulatory Peritoneal Dialysis; Mix Group = dialysis modality changed in 2014; FYD = full year on dialysis; Fig 1B: Classification of transplantation patients. We included only the first received kidney transplantation in the study period of 2012–2014. * Excluded patients are not represented in the figure.

From all adults (≥19 years) in the Vektis claims database who had at least one health insurance claim related to RRT we included those patients on chronic RRT and excluded those with incidental use (e.g. acute dialysis) or unjustified (erroneous) claims. Dialysis patients were selected using health claims in the year 2014 and kidney transplant patients were identified using claims in the period January 1, 2012 to December 31, 2014. We differentiated 7 RRT modalities: (1) in-centre haemodialysis (CHD), (2) home haemodialysis (HHD), (3) continuous ambulatory peritoneal dialysis (CAPD), (4) automated peritoneal dialysis (APD), (5) multiple dialysis modalities within a year (Mix), (6) living kidney donor transplant recipients and (7) deceased kidney donor transplant recipients (Fig 1A and 1B, see also S2 File). After classification, we validated the number of patients per modality in an external database, the Dutch Renal Registry (Renine), that serves as gold standard because of its complete coverage of chronic RRT. Correspondence between the two databases was high (93.8–99.1%, see S3 File).

### Cost variables

We estimated healthcare costs by using registered health claims (reimbursement data). Costs were distinguished according to different healthcare components. First, costs directly related to RRT, based on diagnosis-related group codes (DRG’s), were identified and included all costs of the dialysis procedure (including access surgery and hospitalization for access surgery), the kidney transplant (including donor expenses) as well as the pre- and post-transplant care. More specifically, RRT related costs include all medications used during dialysis (e.g. EPO, phosphate binders), staff costs, including physician fees, laboratory assessments and other diagnostics as included in RRT clinical guidelines (e.g. chest X-ray). Also, equipment and devices needed, e.g. dialysis machines for home dialysis, is included here. Finally, overhead costs, e.g. for water and energy are included. Second, non-RRT costs were defined as all remaining in- and outpatient DRG costs not directly related to RRT, such as primary care, mental healthcare, medication, medical devices, transportation, healthcare costs incurred abroad and other healthcare costs. These non-RRT costs may incur dialysis related costs as well, e.g. transportation costs to and from the dialysis center, but these costs cannot be attributed with 100% certainty to RRT.

Cost data are provided as annual costs, averaged over all patients in a specific modality group. To provide meaningful cost estimates for patients that are not a full year on RRT, e.g. incident and deceased patients, we calculated average 4-week healthcare costs for these patient subgroups.

### Statistical analysis

Descriptive statistics (age, gender and co-morbidity) are presented per treatment modality. The presence of comorbidities was based on medication (see S4 File). Healthcare costs are presented as mean with standard deviation. The average annual costs of haemodialysis patients were calculated depending on weekly dialysis frequency. To test for statistical significance of differences in healthcare expenditure between groups (lower and higher frequency of haemodialysis; donor source), we applied the non-parametric Wilcoxon-Mann-Whitney test, as cost data were non-normally distributed. Finally, healthcare costs were calculated per treatment state. To accommodate for differences in total treatment time between incident, full year on dialysis (FYD) and deceased patients, we calculated the cost of 4 weeks of treatment (4-week costs) per treatment state, as the sum of yearly costs divided by total treatment time (TTT) in days * 28 days. All costs are reported in euros (1 euro = 1.11454 US dollar–exchange rate of 31^th^ of July 2019) and converted to the year 2014 according to the Dutch Consumer Price Index (2012 to 2014: 1.035; 2013 to 2014: 1.010) [15]. All analyses were conducted using SAS, version 9.4 (SAS Institute Inc., Cary, NC).

## Results

### Patient characteristics by RRT modality

Overall, 7,827 persons could reliably be attributed to one of seven RRT modalities in 2014 (Table 1). Of these, 6,876 were dialysis patients and 951 patients received a kidney transplant. On average, HHD patients and transplant recipients were younger and had fewer comorbidities than patients on other modalities, whereas more males than females received RRT.

**Table 1:** Patient characteristics per RRT modality (>75% TTT^a^) in the year 2014.

	Dialysis patients					Kidney transplant recipients	
	On Haemodialysis (HD)		On Peritoneal dialysis (PD)		Mix between dialysis treatments ^a^	Performed Transplants ^c^	
	CHD ^b^	HHD ^b^	APD ^b^	CAPD ^b^	Mix Group ^b^	Deceased donor	Living donor
N (%)	5594 (81%)^d^	187 (3%)	431 (6%)	411 (6%)	253 (4%)	441 (46%) ^e^	510 (54%)
Age (mean, SD)	69.6 (13.9)	58.3 (13.5)	65.7 (14.3)	69.0 (13.1)	63.3 (14.9)	57.0 (12.6)	50.7 (13.7)
Gender (% men)	59%	65%	62%	61%	66%	63%	60%
Nr. of comorbidities (mean, SD)	1.0 (0.9)	0.7 (0.8)	1.1 (0.9)	1.2 (0.9)	1.0 (0.9)	1.0 (0.8)	0.7 (0.8)

^a^ TTT = Total Treatment Time in year 2014 applies to dialysis patients only. RRT modality groups are exclusive, implying that patients are only part of one ‘stable’ RRT modality.

^b^ CHD = Centre Haemodialysis; HHD = Home Haemodialysis; APD = Automated Peritoneal Dialysis; CAPD = Continuous Ambulatory Peritoneal Dialysis; Mix Group = dialysis modality changed in 2014.

^c^ We included only patients with a first kidney transplantation in 2014 in the study period of 2012–2014.

^d^ Reading example: 81% of the dialysis patients were categorized as CHD patients.

^e^ Reading example: 46% of the kidney transplant recipents received a decased donor kidney.

### Annual healthcare costs per dialysis modality

Table 2 shows average annual healthcare costs in 2014 of FYD patients by modality. RRT costs ranged from €61,025 for CAPD patients to €76,531 for Mix group patients. The vast majority of costs was related to the dialysis itself, with relatively small amounts for pre-transplant procedures and dialysis access, but not in the Mix group. This group experiences by definition a change between modalities necessitating costs for access procedures. Three non-RRT healthcare components stand out with relatively high amounts, i.e. hospital costs, medication and transportation costs. Hospital costs unrelated to RRT for patients in the Mix group (€16,286) were much higher than in other groups. The third most expensive cost item was medication (outside the hospital) with the lowest costs for CAPD (€3,939) and, again, highest costs for the Mix group (€4,690). Transportation costs (taxi costs) were highest in CHD patients (€5,455), while home dialysis patients (HHD and (C)APD) had almost ten-fold lower expenditure, obviously related to the less frequent hospital visits of these groups. RRT related costs of HHD were in the same order of magnitude as costs of CHD, this is related to the possibility to use individual nursing assistance at home for HHD patients.

**Table 2:** Average annual healthcare costs per dialysis modality, for FYD patients (Full Year on Dialysis) (€ 2014).

	Haemodialysis (HD)						Peritoneal dialysis (PD)								
	CHD ^a^ (n = 3,430)			HHD ^a^ (n = 174)			CAPD ^a^ (n = 198)			APD ^a^ (n = 282)			Mix Group ^a^ (n = 125)		
	Mean (Std Dev)		% users	Mean (Std Dev)		% users	Mean (Std Dev)		% users	Mean (Std Dev)		% users	Mean (Std Dev)		% users
Dialysis modality	€ 69,887	(7,274)	100%	€ 71,409	(5,645)	100%	€ 60,084	(3,693)	100%	€ 73,437	(3,620)	100%	€ 73,055	(10,002)	100%
Dialysis access	€ 1,645	(2,885)	42%	€ 1,187	(2,420)	36%	€ 529	(1,530)	15%	€ 385	(1,475)	12%	€ 3,137	(3,568)	64%
Pre-transplant procedures	€ 201	(1,379)	11%	€ 239	(791)	19%	€ 413	(1,805)	20%	€ 392	(1,468)	20%	€ 339	(1,186)	22%
**Total RRT costs**	**€ 71,734**	**(8,106)**	**100%**	**€ 72,834**	**(6,338)**	**100%**	**€ 61,025**	**(4,644)**	**100%**	**€ 74,215**	**(4,152)**	**100%**	**€ 76,531**	**(10,747)**	**100%**
Hospital (no RRT)	€ 8,563	(13,813)	93%	€ 5,785	(7,775)	93%	€ 9,115	(22,633)	90%	€ 7,611	(13,401)	91%	€ 16,286	(21,181)	99%
Primary care	€ 395	(606)	98%	€ 340	(520)	97%	€ 351	(437)	97%	€ 346	(508)	99%	€ 446	(756)	98%
Mental health care	€ 236	(2,630)	5%	€ 13	(78)	3%	€ 164	(1,617)	3%	€ 87	(601)	4%	€ 414	(3,239)	9%
Medication ^b^	€ 4,325	(3,395)	98%	€ 4,277	(4,081)	99%	€ 3,939	(3,383)	98%	€ 4,382	(6,199)	99%	€ 4,690	(6,392)	99%
Medical devices	€ 911	(1,787)	70%	€ 2,808	(2,068)	90%	€ 1,726	(1,535)	95%	€ 1,991	(1,515)	95%	€ 2,684	(2,955)	96%
Health care abroad	€ 171	(846)	9%	€ 355	(917)	21%	€ 1	(17)	2%	€ 202	(2,266)	2%	€ 12	(96)	2%
Transportation	€ 5,455	(5,499)	96%	€ 570	(973)	49%	€ 504	(1,015)	41%	€ 461	(964)	34%	€ 2,856	(2,633)	87%
Other	€ 827	(4,007)	20%	€ 69	(489)	13%	€ 739	(3,785)	12%	€ 639	(3,668)	13%	€ 1,914	(5,756)	27%
**Total average annual costs**	**€ 92,616**	(21,500)	**100%**	**€ 87,051**	(12,648)	**100%**	**€ 77,566**	(27,237)	**100%**	**€ 89,932**	(18,890)	**100%**	**€ 105,833**	(30,239)	**100%**

^a^ CHD = Centre Haemodialysis; HHD = Home Haemodialysis; APD = Automated Peritoneal Dialysis; CAPD = Continuous Ambulatory Peritoneal Dialysis; Mix Group = dialysis modality changed in 2014.

^b^ Medication is all medication distributed by pharmacy outside the hospital. Other types of costs, i.e. dialysis modality and hospitalization may also include medication costs. In the Netherlands, inpatient medication is part of the DRG and can therefore not be detected as a separate expenditure in claims data.

### Annual health care costs by dialysis frequency

A reliable dialysis frequency pattern could be established for 3412 out of 3604 FYD haemodialysis patients (Fig 1). Only 8% of these patients dialyzed ≥4 times/week. Of these, the vast majority dialyzed 4 or 5 times/week while 19 patients (7%) received ≥6 sessions/week.

Table 3 shows that frequent users had higher average costs related to RRT (€88,200) in comparison with less frequent users (€69,744). Hospital costs unrelated to RRT did not differ between these groups. Primary care, medical devices, healthcare abroad and transportation costs did however differ between the two groups (although with small differences in euros). Overall, cost differences between the high- and normal-intensity haemodialysis patients amounted to €19,122, in favor of those who dialyze ≤3 times/week.

**Table 3:** Total average annual costs (€) depending on frequency of haemodialysis, for FYD patients (Full Year on Dialysis) only.

	≤ 3 times per week (n = 3,142)			≥4 times per week (n = 270)		
	Mean (Std Dev)		% users	Mean (Std Dev)		% users
N	3142			270		
Total RRT costs*	**€ 69,744**	**(5,067)**	**100%**	**€ 88,200**	**(10,985)**	**100%**
Hospital costs not related to RRT	€ 8,301	(13,425)	92%	€ 8,818	(13,968)	95%
Primary care*	€ 396	(607)	98%	€ 335	(564)	99%
Mental health care	€ 244	(2,736)	5%	€ 47	(469)	4%
Medication^a^	€ 4,292	(3,410)	98%	€ 4,349	(3,483)	99%
Medical devices*	€ 907	(1,734)	70%	€ 1,714	(2,274)	77%
Health care abroad*	€ 169	(831)	9%	€ 213	(727)	13%
Transportion*	€ 5,219	(5,276)	96%	€ 5,186	(7,516)	77%
Other	€ 848	(4,074)	20%	€ 379	(2,803)	17%
Total average annual costs*	**€ 90,119**	**(19,981)**		**€ 109,241**	**(23,295)**	

*Significance at p<0.05 two-sided.

^a^ Medication is all medication distributed by pharmacy outside the hospital. Other types of costs, i.e. dialysis modality and hospitalization may also include medication costs. In the Netherlands, inpatient medication is part of the DRG and can therefore not be detected as a separate expenditure in claims data.

### 4-week healthcare costs per treatment state and dialysis modality

**Fig 2:**
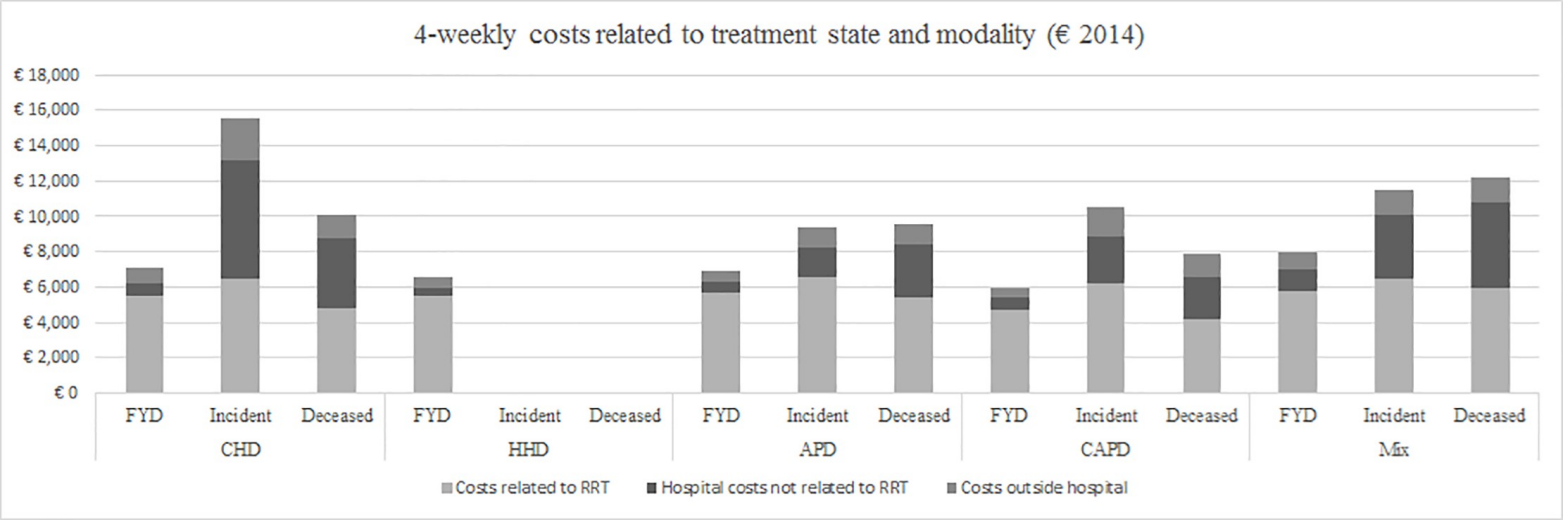
4-week average mean healthcare costs related to treatment states, per modality. Note: FYD = full year on dialysis; Incident = incident patients starting treatment in 2014; Deceased = patients who died in 2014; CHD = Centre Haemodialysis; HHD = Home Haemodialysis; APD = Automated Peritoneal Dialysis; CAPD = Continuous Ambulatory Peritoneal Dialysis; Mix Group = dialysis modality changed in 2014.

Fig 2 shows the 2014 average 4-week healthcare costs of 3 dialysis subgroups (indicated as “treatment states”): (1) FYD patients, (2) incident patients and (3) deceased patients. Hence, independent of the TTT or period alive, Fig 2 enables a comparison of the five dialysis modalities per treatment state while including all healthcare spending over the year 2014. Incident and deceased HHD patient numbers were too low in number to allow meaningful analysis. Fig 2 confirms that most costs of FYD are related to RRT. The highest 4-week expenditures (€15,560) were made for patients starting CHD. For all dialysis modalities, incident and deceased patients had high non-RRT related costs, while FYD patients had low non-RRT related costs. Highest 4-week costs are associated with patients starting CHD and lowest 4-week costs were registered for stable CAPD patients.

### Annual health care costs of kidney transplantation

Table 4 shows that the average annual healthcare costs in the year *of* transplantation are high (€85,127) and comparable to annual healthcare costs of dialysis patients (€77.566 - €105.833, see Table 2). The annual healthcare costs decline with time after surgery, with total annual healthcare costs of €29,612 in the first and €15,018 in the second year after successful transplantation.

**Table 4:** Total average annual healthcare costs per phase of transplantation.

	Year of transplantation			First year^a^ after transplantation			Second year after transplantation			Second year after successful transplantation		
N	2,829			1,825			911			806		
	Mean (Std Dev)		% users	Mean (Std Dev)		% users	Mean (Std Dev)		% users	Mean (Std Dev)		% users
Preparatory research	€ 1,954	(7,911)	45%	€ 100	(857)	5%	€ 29	(223)	4%	€ 19	(205)	2%
Transplant operation	€ 22,748	(11,267)	100%	€ 1,150	(4,454)	9%	€ 242	(2,374)	2%	€ 55	(731)	1%
Guidance^b^	€ 474	(1,154)	17%	€ 106	(474)	7%	€ 99	(394)	8%	€ 108	(413)	9%
After care	€ 6,803	(9,641)	74%	€ 4,396	(6,438)	85%	€ 1,831	(2,777)	82%	€ 1,817	(2,566)	86%
Donor expenses	€ 2,608	(5,466)	23%	€ 246	(1,623)	5%	€ 6	(102)	1%	€ 5	(84)	1%
Dialysis procedure (incl. access)	€ 28,020	(26,007)	74%	€ 5,048	(15,442)	21%	€ 3,635	(14,738)	10%	€ 0	(0)	0%
**RRT related costs**	**€ 62,607**	**(31,064)**	**100%**	**€ 11,046**	**(18,815)**	**92%**	**€ 5,842**	**(15,612)**	**90%**	**€ 2,004**	**(2,647)**	**90%**
Hospital (no RRT)	€ 9,550	(17,670)	97%	€ 7,242	(23,245)	87%	€ 5,698	(16,520)	86%	€ 4,694	(12,782)	86%
Primary care	€ 246	(328)	99%	€ 276	(377)	100%	€ 285	(506)	100%	€ 281	(522)	100%
Mental health care	€ 147	(1,597)	6%	€ 263	(3,392)	6%	€ 195	(1,306)	6%	€ 204	(1,380)	6%
Medication^c^	€ 9,227	(6,458)	100%	€ 8,776	(5,422)	100%	€ 6,536	(4,253)	100%	€ 6,587	(4,258)	100%
Medical devices	€ 678	(1,395)	62%	€ 616	(1,375)	51%	€ 664	(1,640)	49%	€ 648	(1,671)	47%
Health care abroad	€ 215	(1,243)	10%	€ 59	(710)	4%	€ 74	(1,128)	5%	€ 52	(1,143)	4%
Transportation	€ 2,237	(3,504)	70%	€ 1,041	(2,307)	47%	€ 608	(1,999)	26%	€ 343	(1,099)	21%
Other	€ 220	(1,808)	13%	€ 293	(2,471)	12%	€ 256	(2,969)	10%	€ 180	(2,641)	11%
**Total average annual costs**	**€ 85,127**	**(39,679)**	**100%**	**€ 29,612**	**(34,099)**	**100%**	**€ 20,156**	**(26,571)**	**100%**	**€ 15,018**	**(16,186)**	**100%**

^a^ A ‘year’ refers to a calendar year. For example, when the transplantation has taken place in 2014, the first year after transplantation was 2015

^b^ Guidance are costs for the process before the transplantation

^c^ Medication is all medication distributed by pharmacy outside the hospital. Other types of costs, i.e. dialysis modality and hospitalization may also include medication costs. In the Netherlands, inpatient medication is part of the DRG and can therefore not be detected as a separate expenditure in claims data.

At the beginning of the second year after transplantation, 911 out of 968 patients transplanted in 2012 were alive, however, 105 of these patients had experienced graft failure, as appeared from claims indicating another kidney transplantation or a return to dialysis. Of the patients with functioning graft (Table 4, last column, n = 806), average annual healthcare expenditure was €5,139 lower than the average second year costs of all patients alive, irrespective of graft functioning.

### Annual healthcare costs of patients according to donor source

Over a 3-year period (2012–2014) 1,554 patients received a kidney transplant from a living kidney donor and 1,275 patients obtained a kidney from a deceased person (Table 5). RRT related costs were most prominent in both groups, varying from 72% of all healthcare costs (living donor) to 75% of costs (deceased donor). Absolute RRT related costs were almost €22,000 higher in deceased donor kidney recipients. This was mostly due to higher dialysis and transplant surgery related costs in recipients of a deceased donor kidney. On average, a transplant from a deceased donor resulted in higher costs in all healthcare components, except donor expenses, compared to receiving a transplant from a living donor. As a result, the total costs related to a deceased donor kidney transplant were substantially higher (€99,450 per year) compared to those of a transplant with a living donor kidney (€73,376).

**Table 5:** Total average annual costs of transplantation (year 0) by source of kidney donor (€).

	Deceased donor			Living donor			Cost	
	Mean (Std Dev)		% users	Mean (Std Dev)		% users	difference	
N	1275			1554				
Preparatory research	€ 578	(2,254)	29%	€ 3,083	(10,343)	58%	€ 2,505	*
Transplant operation	€ 27,034	(12,083)	100%	€ 19,232	(9,162)	100%	-€ 7,802	*
Guidance	€ 256	(847)	11%	€ 652	(1,329)	22%	€ 396	*
After care	€ 7,485	(11,034)	73%	€ 6,244	(8,287)	75%	-€ 1,241	
Donor expenses	€ 28	(254)	2%	€ 4,725	(6,663)	40%	€ 4,697	*
Dialysis procedure (incl. access)	€ 39,223	(24,281)	94%	€ 18,828	(23,674)	57%	-€ 20,394	*
**Total RRT costs**	**€ 74,604**	**(28,121)**	**100%**	**€ 52,764**	**(29,889)**	**100%**	**-€ 21,839**	*
Hospital (no RRT)	€ 10,571	(18,771)	98%	€ 8,712	(16,672)	96%	-€ 1,859	*
Primary care	€ 249	(352)	100%	€ 243	(307)	99%	-€ 6	
Mental health care	€ 210	(2,122)	7%	€ 96	(972)	5%	-€ 114	
Medication^a^	€ 9,442	(6,143)	100%	€ 9,051	(6,703)	100%	-€ 391	*
Medical devices	€ 743	(1,376)	70%	€ 625	(1,409)	56%	-€ 118	*
Health care abroad	€ 317	(1,578)	11%	€ 131	(868)	9%	-€ 186	
Transportation	€ 3,001	(3,934)	76%	€ 1,611	(2,966)	64%	-€ 1,390	*
Other	€ 313	(2,092)	14%	€ 144	(1,533)	12%	-€ 169	*
**Total average annual costs**	**€ 99,450**	**(36,036)**	**100%**	**€ 73,376**	**(38,666)**	**100%**	**-€ 26,074**	*

* = significant at P<0.05 two-sided.

^a^ Medication is all medication distributed by pharmacy outside the hospital. Other types of costs, i.e. dialysis modality and hospitalization may also include medication costs. In the Netherlands, inpatient medication is part of the DRG and can therefore not be detected as a separate expenditure in claims data.

## Discussion

Our study using health insurance claims showed high expenses for all dialysis modalities, with annual expenditure ranging between €77,566 for CAPD to €92,616 for CHD and €105,833 for patients of the Mix group. The vast majority of total health care expenses was related to RRT. Patients who dialyzed more frequently had higher overall expenditure because higher RRT related costs were not compensated by lower non-RRT related costs. In the year of kidney transplantation, patients had expenses similar to those on dialysis, but expenses declined steadily in the years post-transplant to €15,018 in the second year for those with a surviving graft after transplantation. Our study found substantial higher expenditure for those who received a kidney from a deceased donor compared to a living donor.

The fact that dialysis treatment is expensive confirms findings of both older [6] and more recent [7–12] studies. Our study also confirms that expenses for CAPD are the lowest among the dialysis modalities [16, 17]. Our study does not confirm observations from small cohort studies showing that haemodialysis patients who dialyze more frequently have lower overall costs [18–20]. The higher RRT related costs of patients who dialyze ≥4 times/week appeared not to be compensated by lower expenses for other healthcare use. Here, we cannot exclude the possibility that selection bias plays a role, with patients in more serve condition qualifying for more intensive dialysis, leaving open the option that their costs would have been higher should they have received regular dialysis 3 times per week. As our study only concerns costs, and not health outcomes (more intensive dialysis is reflected in better patient outcomes, such as mortality and physical health [21]), a separate cost-effectiveness analysis comparing more and less intensive dialysis treatment would be needed to find out whether additional costs are balanced by better outcomes. Such a cost-effectiveness analysis should be undertaken from a societal perspective, to include types of costs that we currently could not address, such as out-of-pocket costs of patients and productivity costs related to the patients’ ability to maintain employment.

Expenses for patients of the Mixed group were remarkably higher in many categories of healthcare use, such as access procedures and medication. In particular, non-RRT related hospital care expenditure in this group was higher than in patients stable on one dialysis modality. This suggests that the switch between dialysis modalities may not only be rooted in therapy failure itself, but also in the occurrence of other diseases that prevent continuation of the initial modality and that are associated with higher non-RRT costs in itself. Indeed, a study from the US [22] showed infections and cardiovascular diseases, mainly fluid overload, to be the most important causes of a switch from peritoneal dialysis to haemodialyis. Also, patient characteristics such as higher BMI and having diabetes were found to be associated with a switch between dialysis modalities. We only had access to few background characteristics of patients, such as age, sex and the number of comorbidities. Patients switching between modalities were somewhat younger than most other dialysis groups, except HHD, but had similar number of comorbidities. Patient groups may have also differed with regard to other, non-measured, predictors of switching between modalities.

Our study shows a clear cost advantage of transplantation using living donor kidneys compared to deceased donor kidneys, despite additional health expenses for the donor. There was a large difference in dialysis costs, i.e. the dialysis costs were higher for the group who received a kidney from a deceased donor. This is likely related to a substantial proportion of living donor kidney procedures being pre-emptive in the Netherlands. Indeed, 33% of patients receiving a graft from a living donor did not receive dialysis at all during the year of transplantation. Other factors possibly related to lower expenses for those who receive a living donor kidney are better survival [23, 24] and less post-operative complications. The latter was confirmed in a recent Japanese study also using health claims data. This study showed longer hospitalization and more urinary tract infections, sepsis and pneumonia in recipients of post-mortal donor kidneys [25]. One further explanation for higher overall costs in patients receiving a deceased kidney organ is the more frequent occurrence of delayed graft function, associated with the need for short term post-transplant dialysis [26].

Annual costs decline fast in the first and second year after transplantation, with medication costs being the highest (30–32% of total cost) component of expenses. These figures include those for non-successful transplantation (n = 105), hence for patients with graft failure who had to return to dialysis and patients who died. Combined with the cost advantages of pre-emptive transplantation as discussed above and the ongoing shortage of deceased donor organs, this stresses the importance of discussing and exploring the possibility of a living donor transplantation in pre-dialysis patients. Recently, the Dutch Parliament accepted a change from opting-in for transplantation after death to an opt-out system, that is expected to increase the number of deceased donor transplantations in the future. Given the small cost differences between pre-emptive transplantation and deceased donor transplantation, relative to the large cost-differences between any transplantation and dialysis, every transplantation is expected to contribute to a decrease in costs of RRT.

Following from the source of the data, being insurance claims, we have to face several limitations of our data. First, we have no guarantees that all transplantation related costs of living donors were registered on the ID of the recipient, with the possible consequence of underestimating the costs of living donors. Second, the donor costs of deceased donors are not at all part of the claims data, as these are reimbursed outside the basic health insurance. Moreover, societal costs (e.g., incapability to work) and out-of-pocket costs were not part of this study, whereas the limited time frame of two post-transplant years prevents us from predicting cost levels of former transplantation patients in later years. However, as annual costs decline fast after transplantation and are lowest in those without graft failure, there is no reason to expect that the cost difference between dialysis and having a functioning transplant kidney will fade out in later years.

One general limitation to studying costs in terms of expenditure is that expenditure is only to be seen as an administrative proxy for real costs, implying that it is unknown whether these costs reflect “true” resource use (both staff and material resources) needed to provide healthcare to these patients. Another important limitation is that we have not related observed differences in expenditure between modalities to differences in patient characteristics, as we only had limited information on background characteristics of patients. A previous study on dialysis patients shows that age is associated with expenditures where elderly dialysis patients often have lower healthcare costs than younger dialysis patients [27]. This knowledge is important when interpreting the results of our study, where patients in the CHD and CAPD groups were somewhat older than in other dialysis groups. At least, the number of comorbid diseases were similar across groups. Other important characteristics, such as frailty, were not known to us. Summing up, it is likely that differences in expenditures between modalities are related to (non-)observed differences in patient characteristics and not due to modality characteristics per se. We feel however that in this specific study it is not the statistical significance of cost differences between groups that is of primary interest, but more the actual differences in expenses, as these are meaningful for health insurers and health policy makers.

Strengths of our study include the national coverage (>99% of population) of our data, the inclusion of all RRT modalities, the inclusion of all healthcare use covered by the Health Insurance Act and not only the part that is related to RRT, the good validation of the data with another national database of RRT patients, and data coverage over a three-year period. We used a rigorous approach of classifying patients into 1 of 5 dialysis groups and 2 different transplantation groups, and excluded all patients with erroneous or temporary RRT claims, as well as patients for whom we had diverging information, e.g. with regard to the weekly number of dialysis procedures. For the included patients we have high levels of confidence that expenditure figures can be attributed correctly to the dialysis modality. Furthermore, as a consequence of our assignment of patients to different treatment groups, we were able to show a clear picture of cost differences between stable patients using different dialysis modalities. Because we present cost data also for periods of 4-weeks, we could show that incident and deceased patients have much higher non-RRT related costs than patients who are stable on one dialysis modality.

Our data stress the fact that CAPD has a clear cost advantage compared to other home-based therapy and CHD. Starting as many patients as possible on CAPD could reduce the high budget impact of RRT to a certain extent. Approximately 1750 patients start dialysis treatment annually in the Netherlands, of whom 20% start PD (CAPD and APD aggregated) [3]. It is clear that not all patients are suitable candidates to start with PD, and that a mismatch between the requirements of the dialysis modality and the patient’s capacity may have detrimental effects, both on costs and outcomes. However, historically, much higher numbers have started with peritoneal dialysis in the Netherlands, and even today, center differences in patients starting with PD range from 1 to 46%. This leaves us to conclude that advantages of several million euros annually could be reached if a start on PD would be considered more often. Obviously, careful pre-dialysis education followed by shared decision making should ensure that only patients who are fit for PD are selected to start with this modality. Furthermore, recent research suggested that the utmost should be done to prevent progression of kidney diseases to kidney failure, and that prevention holds the promise of spending less health care resources on RRT [28, 29].

We conclude that annual healthcare expenditures of RRT patients are high, while showing relevant differences between dialysis modalities at the same time. Frequent haemodialysis patients have higher RRT related expenditure compared to patients with regular dialysis frequency with similar non-RRT costs. Dialysis patients of the Mix group have the highest annual expenditure, in particular in the RRT unrelated costs. Annual healthcare costs in the year of transplantation are high, but decline fast in the years after transplantation. Living donor kidney transplantation incurs lower costs compared to transplantation with a deceased donor kidney. Therefore, our results indicate that the current practice in the Netherlands, where (pre-emptive) living donor procedures are actively encouraged by nephrologists, is associated with cost advantages in both the short and longer term. At the same time, budget impact of RRT could be diminished to a certain extent when more patients would start treatment with home-based therapies, especially CAPD.

## Supporting information

S1 File (DOCX)

S2 File (DOCX)

S3 File (DOCX)

S4 File (DOCX)
