# Supplementary material for: Healthcare costs of patients on different renal replacement modalities – Analysis of Dutch health insurance claims data
Source: PLoS One. 2019 Aug 15;14(8):e0220800. doi: 10.1371/journal.pone.0220800 (PMC6695145; doi:10.1371/journal.pone.0220800)
Supplement: S1 File — (DOCX) [file pone.0220800.s001.docx]

# Supporting Information S1 File. Request for mandate -

# With regard to the processing of personal data for which the health insurers are responsible

| 1. | Aim of the study | Within the project The Dutch Kidney Atlas: Vektis data will be analysed on health care and costs of patients with kidney disease. This information will be published on a website. In addition, scientific paper will be written on this topic. |
| --- | --- | --- |
| 2. | Grant | Dutch Kidney Foundation |
| 3. | Researchers | Project Dutch Kidney Atlas: Researchers Academic Medical Centre; Onderzoekscentrum Ketenzorg chronische ziekten Isala, and Nefrovisie. |
| 4. | Results of the study | Health care use of patients with kidney disease, as well as their comorbidities, medication use and other treatments, and outcomes |
|  |  | See also the appendix |
| 5. | What is the value of this project for insurance companies or health care providers? | The results may add to quality of care for patients with kidney disease. The scientific papers provide transparency. The society also asks for publicity of these kind of results. |
| 5a | Is there feedback of the results to the health insurers? | The Dutch Kidney Atlas will be published on a website. These data can be used by health insurers. |
| 6. | Are all heath insurance data used for the analyses? | Yes |
| 7. | Are these analyses performed once or repeated over time? | Repeated (aimed at publishing the Dutch Kidney Atlas once a year) |
| 8. | Is this agreement in correspondence to other projects? If so, which projects? | Yes: project 6-259 'Costs for renal replacement therapy'. |
| 9. | Advice of Vektis with regard to this project? | Vektis advice: BCVU should also provide permission |
| 10. | Contactperson | Niels Hoeksema ([n.hoeksema@vektis.nl](mailto:n.hoeksema@vektis.nl) of 06-10179269/030-8008358) of Michiel ten Hove ([m.ten.hove@vektis.nl](mailto:m.ten.hove@vektis.nl) of 030-8008359) |

**Appendix**

## 6-258 Dutch kidney atlas

| **Output personal data** | | **Points for attention** | |
| --- | --- | --- | --- |
| **Output:** |  | Date | |
|  |  | Date | 18 February 2016 |
|  |  | Aim and law | |
|  |  | **Aim:**   - Improvement of quality of care. - Scientific papers.   **Lawful basis for processing:**   - ZVW: art. 88 en 89 - WLZ: art. 9.1.3 - WBP: art.23 | |
|  |  | Vektis restrictions | |
|  |  | - The individual data will not be provided: the data are only accessible within the secured environment of Vektis. The researchers should sign a confidentiality statement. - Only aggregated results could leave the Vektis building, and identification of individuals is not possible. | |
|  |  | Restrictions for researchers | |
|  |  | - The data can only be used for the described goals. - The data that will be published should not include information that could trace individuals, health providers or insurance companies if they did not provide this consent. - The publications should include a reference of the source data. - The research should be performed within the secured environment of Vektis. | |
|  |  | Researchers: | |
|  |  | - Project Dutch Kidney Atlas | |
|  |  | Once/repeated | |
|  |  | Analyses repeated over time (atlas will be published each year) | |
|  |  | Responsibel: | |
|  |  | Heath insurers | |
|  |  | Outside EU? | |
|  |  | No | |
