# Supplementary material for: Healthcare costs of patients on different renal replacement modalities – Analysis of Dutch health insurance claims data
Source: PLoS One. 2019 Aug 15;14(8):e0220800. doi: 10.1371/journal.pone.0220800 (PMC6695145; doi:10.1371/journal.pone.0220800)
Supplement: S2 File — (DOCX) [file pone.0220800.s002.docx]

# Supporting Information S2 File: Elaboration of terms and concepts of Fig 1 (Classification of RRT modalities)

Dialysis patients
Patients were selected with at least one insurance claim relating to dialysis in 2014. After exclusion of newly transplanted patients in 2014 (n=970) and patients with missing information (n=4), the remaining 7,881 patients were classified into five dialysis groups (Fig 1, Step 1). Patients were attributed to a dialysis modality if they were *stable* on one modality for ≥75% of their total treatment time (TTT), which is defined as the number of days in 2014 with a RRT related claim. Patients with less than 75% of their TTT on one dialysis modality were allocated to the *Mix* dialysis group, similar to methods used by Couillerot-Peyrondet et al. [9]. All dialysis patients were further categorized into three subgroups (also indicated as “treatment states”; Fig 1, Step 2): (1) patients who were on dialysis the full-year (FYD), (2) incident patients starting treatment in 2014, and (3) patients who died in 2014 (Fig 1). Incident patients were on treatment for a minimum of 45 days and did not decease. Patients who were not allocated to one of the treatment states were excluded (n=1,005). Finally, patients with FYD hemodialysis were subdived into dialysis frequency ≤3 times (n=3142) or ≥4 times (n=270) per week (Fig 1, Step 3). Patients with a high variation in dialysis frequency were excluded from this subanalysis (n=192).

## Kidney transplant recipients

We identified patients receiving a kidney transplant in the period 2012-2014 by using surgery claims for kidney transplantation. In contrast to dialysis, claims data over a longer period (2012-2014) were used to analyse the development in costs over the year preceding the transplant, the year of transplantation and years post-transplantation. We subdivided patients into those receiving a kidney from a living versus a deceased donor (Fig2). If a patient received more than one kidney transplant, only the first transplant was taken into account. The date of first transplant was used to calculate the average annual healthcare costs per transplant phase: the year before transplantation, the year of transplantation, and the first and second year after transplantation. For instance, second year post transplant data were only available for patients transplanted in 2012. We also differentiated between patients in the second year post transplantation with and without a functioning graft.
