# Supplementary material for: Healthcare costs of patients on different renal replacement modalities – Analysis of Dutch health insurance claims data
Source: PLoS One. 2019 Aug 15;14(8):e0220800. doi: 10.1371/journal.pone.0220800 (PMC6695145; doi:10.1371/journal.pone.0220800)
Supplement: S3 File — (DOCX) [file pone.0220800.s003.docx]

# Supporting Information S3 File: Validation of the number of patients as identified by the Dutch claims data (Vektis database)

Table 1 of S3 File: Comparison of the number of identified patients on different dialysis modalities (e.g. chronic centre haemodialysis (CHD), chronic home haemodialysis (HHD), continuous ambulatory peritoneal dialysis (CAPD) and automated peritoneal dialysis (APD) on the 1st of January 2014 between the Dutch claims database and the Dutch renal registry (Renine). The Dutch renal registry is considered as the gold standard.^[[1]](#footnote-1)^

|  | Dutch claims database | Dutch renal registry | Difference (%) |
| --- | --- | --- | --- |
| HD total | 5478 | 5531 | 1.0 |
| chronic CHD | 5261 | 5310 | 0.9 |
| chronic HHD | 217 | 221 | 1.8 |
| PD total | 919 | 915 | 0.4 |
| CAPD | 401 | 427 | 6.1 |
| APD | 518 | 488 | 6.2 |

Table 2 of S3 File: Comparison of the number of identified kidney transplants performed using living and deceased donors in 2014 between the Dutch claims database and the Dutch renal registry (Renine). The Dutch renal registry is considered as the golden standard.^[[2]](#footnote-2)^

|  | Dutch claims database | Dutch renal registry | Difference (%) |
| --- | --- | --- | --- |
| Transplantation Total | 976 | 967 | 0.9 |
| Living donor | 521 | 505 | 3.2 |
| Deceased donor | 455 | 462 | 1.5 |

1. The numbers differ with the numbers in our study, due to the selection of dialysis patients with claims data during the full year of 2014 in the study (see Figure 1 of main paper). [↑](#footnote-ref-1)
2. The numbers differ slightly with the numbers in our study, due to the exclusion of re-transplantations during the study period. [↑](#footnote-ref-2)
