# Supplementary material for: Healthcare costs of patients on different renal replacement modalities – Analysis of Dutch health insurance claims data
Source: PLoS One. 2019 Aug 15;14(8):e0220800. doi: 10.1371/journal.pone.0220800 (PMC6695145; doi:10.1371/journal.pone.0220800)
Supplement: S4 File — (DOCX) [file pone.0220800.s004.docx]

# Supporting Information S4 File: Selection of comorbidities

Comorbidities were assigned based on the Pharmaceutical Cost Groups (FKGs). The classification of FKGs is based on medication claims and used as indication for a chronic condition. A person is assigned to a FKG by Vektis when the annual use is more than 180 standard daily doses of medication associated with a chronic disease. As part of the risk adjustment system, Dutch insurance companies receive a higher contribution from the Health Insurance Fund for chronically ill people as they have higher predictable healthcare costs in subsequent years compared to healthy people.

Vektis applied the current version (2016) of the FKG list to derive chronic condition groups in the years before. In this study, medication claims of the year 2014 were used to indicate chronic condition groups for the year 2014 according to the FKG classification of 2016:

1. Asthma;
2. Cystic fibrosis/pancreatitis;
3. High Cholesterol;
4. COPD;
5. Crohn's disease/Colitis Ulcerosa;
6. Diabetes type I;
7. Diabetes type II with hypertension;
8. Diabetes type II without hypertension;
9. Depression;
10. Epilepsy;
11. Glaucoma;
12. Grow hormones;
13. Heart disorder;
14. HIV/AIDS;
15. Hormone-sensitive tumours;
16. Hypertension;
17. Cancer;
18. Kidney disease;
19. Brain & Spinal cord disorders and injuries;
20. Neuropathic pain;
21. Parkinson's disease;
22. Psychosis, Alzheimer's and Addiction;
23. Rheumatism;
24. Thyroid disorders;
25. TNF alpha-inhibitors;
26. Transplantation;
27. Chronic pain exclusive opioid;
28. Pulmonary (arterial) hypertension;
29. Psoriasis;
30. Multiple sclerosis (MS);
31. Auto-immune diseases;
32. Cancer;

A total of 32 FKG groups are defined in the Vektis database, but the number of chronic condition groups that can be derived from the FKG groups is less. First, the FKGs kidney disease and transplantation are the main diagnosis of our study population, which means that these conditions cannot be considered as comorbidity. Second, several restrictions are applied in the classification process in order to avoid double-counting of chronic conditions. For instance, a person assigned to the FKG ‘Psychosis, Alzheimer's and Addiction’ is not allowed to be classified in the FKG ‘Depression’ as well. The same restriction was applied in respiratory diseases, auto-immune diseases, diabetes, cancer and brain & spinal cord disorders. Third, hypertension and high cholesterol are considered as risk factors and cannot be counted as equal to another ‘real’ comorbidity (e.g. cancer). In addition, the distinctiveness of these FKGs is worthless as at least one of the FKGs occurs in >95% of our study population. Therefore, both hypertension and high cholesterol were removed from our chronic condition list. Fourth, the FKG-list of 2016 contains two new FKGs; for grow hormones and TNF-alpha inhibitors. However, it was not possible to differentiate these FKGS with the claims data of 2014. At last, chronic pain (exclusive opioid) was excluded due to the fact that pain usually is a result from a comorbidity.

Based on the considerations above, the following list of chronic conditions is derived from the FKG groups and applied in our study:

1. Respiratory diseases: Asthma & COPD;
2. Auto-immune diseases: Crohn's disease/Colitis Ulcerosa, Psoriasis & Rheumatism;
3. Cystic fibrosis/pancreatitis;
4. Diabetes type I and II;
5. Neuropathic pain;
6. Mental disorders: Psychosis, Alzheimer's and Addiction & Depression;
7. Epilepsy;
8. Glaucoma;
9. Heart disorder;
10. HIV/AIDS;
11. Cancer: Hormone-sensitive tumours & Metastasis
12. Brain & Spinal cord disorders and injuries & Multiple Sclerosis (MS);
13. Parkinson's disease;
14. Pulmonary (arterial) hypertension
15. Thyroid disorders.
